# Equity of PrEP uptake by race, ethnicity, sex and region in the United States in the first decade of PrEP: a population-based analysis

**DOI:** 10.1016/j.lana.2024.100738

**Published:** 2024-04-22

**Authors:** Patrick S. Sullivan, Stephanie N. DuBose, Amanda D. Castel, Karen W. Hoover, Marta Juhasz, Jodie L. Guest, Gordon Le, Shamaya Whitby, Aaron J. Siegler

**Affiliations:** a Rollins School of Public Health, Emory University, Atlanta, GA, USA; b The George Washington University Milken Institute School of Public Health, Washington, DC, USA; c Centers for Disease Control and Prevention, Atlanta, GA, USA; d Saluda Analytics, Budapest, Hungary

**Keywords:** PrEP, Health equity, Sex, Region, PrEP to need ratio, US South

## Abstract

**Background:**

PrEP was approved for HIV prevention in the US in 2012; uptake has been slow. We describe relative equity with the PrEP Equity Ratio (PER), a ratio of PrEP-to-Need Ratios (PnRs).

**Methods:**

We used commercial pharmacy data to enumerate PrEP users by race and ethnicity, sex, and US Census region from 2012 to 2021. We report annual race and ethnicity-, sex-, and region-specific rates of PrEP use and PnR, a metric of PrEP equity, to assess trends.

**Findings:**

PrEP use increased for Black, Hispanic and White Americans from 2012 to 2021. By 2021, the rate of PrEP use per population was similar in Black and White populations but slightly lower among Hispanic populations. PnR increased from 2012 to 2021 for all races and ethnicities and regions; levels of PrEP use were inconsistent across regions and highly inequitable by race, ethnicity, and sex. In all regions, PnR was highest for White and lowest for Black people. Inequity in PrEP use by race and ethnicity, as measured by the PER, grew early after availability of PrEP and persisted at a level substantially below equitable PrEP use.

**Interpretation:**

From 2012 to 2021, PrEP use increased among Americans, but PrEP equity for Black and Hispanic Americans decreased. The US South lagged all regions in equitable PrEP use. Improved equity in PrEP use will be not only just, but also impactful on the US HIV epidemic; persons most at-risk of acquiring HIV should have the highest levels of access to PrEP. Prevention programs should be guided by PrEP equity, not PrEP equality.

**Funding:**

10.13039/100000002National Institutes of Health, 10.13039/100005564Gilead Sciences.

**Research in context**

**Evidence before this study**

Pre-exposure prophylaxis is effective at reducing the risk of acquiring HIV when taken consistently and correctly. However, in the first decade of its availability for clinical use, uptake has been limited, and data about uptake in specific groups of Americans (e.g., women, Black and Hispanic people) suggest that uptake might be inequitable. The PrEP-to-Need ratio has been developed and reported as a metric of equitable use within specific populations with high risk of acquiring HIV, but systematic comparisons between groups (e.g., Black versus White, female versus male) have not been described.

**Added value of this study**

This study uses commercial pharmacy data to evaluate population-based trends in PrEP equity from 2012 to 2021 in the United States overall and by race, sex, and Census region. The study also proposes the PrEP Equity Ratio, which is constructed as a ratio of two PnR measures, and quantifies the extent of inequity between two groups. The results help to quantify the extent to which some groups of people at risk for HIV – for example, women, Black people, Hispanic people and people in the US South – are under-represented among PrEP users, relative to their epidemic need.

**Implications of all the available evidence**

Group-specific PrEP equity measures (PnR) have raised awareness of the extent to which PrEP use fails to reach the people who need it most in the HIV epidemic. Group-specific PnR measures document that Black, Hispanic, Southern people, and women are underserved relative to their epidemic need. The addition of PrEP Equity Ratios adds another way to depict the trends in equitable PrEP use; the results suggest that the extent to which Black, Hispanic, and females, have been underserved by PrEP is profound, and that PrEP equity has not improved in the past decade. To increase equitable PrEP use and to curb rates of new HIV infections, we must monitor measures of PrEP equity at the programmatic, state, regional and federal levels, and support interventions and implementation strategies that offer promise of getting PrEP to the people who need it most.

## Introduction

Pre-exposure prophylaxis (PrEP) has been proven to be effective in decreasing the risk of acquiring HIV among people with behaviours associated HIV acquisition.^1^^,^^2^ However, uptake of PrEP among people with PrEP indications in the United States (US) has been slow.^3^ To realize the full public health benefits of PrEP, it is important to develop PrEP use metrics and monitor PrEP uptake to understand the success of programs to promote its use. Ultimately, the impact of PrEP on reducing new HIV infections will depend both on the number of people with indications who are using PrEP, and the extent to which the people using PrEP are the people with the highest risks for acquiring HIV. For example, in the US, rates of new HIV infections are higher among Black people and Hispanic people relative to White people and are higher among people who live in the US South relative to people who live in other areas of the country, an indication of how persistent inequality in access has led to further inequities in PrEP uptake.^4^ The prevention benefits of PrEP will be maximized by achieving levels of PrEP use in Black, Hispanic, and Southern people that are in proportion to their burdens of HIV infections.

Monitoring PrEP utilization in the US is complicated by the lack of a national system for providing and tracking PrEP prescriptions. Numerous studies have reported the extent of PrEP uptake in specific clinical settings (e.g., in LGBT-focused health clinics^5^ or other closed healthcare systems^6^), and others have analysed data on the proportions of people responding to surveys who report PrEP use.^7^ These estimates are important, but suffer from biases related to access to healthcare, participation in surveys, and response to questions about PrEP use. We and others have used prescription data from US retail and mail order outlet pharmacies to develop estimates of PrEP use^8^; such estimates are also biased by missing information from closed healthcare systems (e.g., Kaiser Permanente, Veteran's Administration). Most published estimates express use as proportions of respondents to surveys, or rates of use per unit of population size for the geographic unit of analysis.

Better ways are needed to examine the equitable uptake of PrEP in populations. Ideally, communities with higher risk of acquiring HIV should have higher uptake of PrEP – i.e., utilisation in proportion to risk. The PrEP-to-Need ratio (PnR) represents a metric of PrEP equity that expresses use relative to new HIV diagnoses,^9^ and has been reported for US states and counties, and by age, sex and race/ethnicity. Here, we report trends in PrEP uptake overall and by US region, race and ethnicity, and sex in the US to describe overall changes in PrEP use in the first decade since PrEP regulatory approval.^10^, ^11^, ^12^, ^13^, ^14^, ^15^, ^16^ We also present trends in race and ethnicity- and sex-specific PnRs using the PrEP Equity Ratio (PER) to characterise the extent to which PrEP use is equitable or inequitable.

## Methods

### PrEP prescriptions

We used commercial pharmacy data (IQVIA, Durham, NC) to identify PrEP prescriptions, using methods previously reported.^8^ These data include information about prescriptions dispensed by more than 90% of retail pharmacies and uses national estimates of prescription fills to estimate PrEP prescriptions for the small percentage of US prescriptions that are not tracked directly by IQVIA. Federal and State PrEP programs are included, to the extent that prescriptions from them are filled through pharmacies. Because the data do not include identifiers, human subjects review was not required.

Briefly, we used an algorithm to differentiate medication use for HIV treatment from use for HIV PrEP by using prescription and diagnoses data. All analyses were conducted in SAS. We included prescriptions for tenofovir disoproxil fumarate/emtricitabine (TDF/FTC) (starting in 2012), tenofovir alafenamide/emtricitabine/(TAF/FTC) after its approval in 2019, and cabotegravir after its approval in 2021. We excluded prescriptions for TDF/FTC,TAF/FTC, and cabotegravir that were provided for other known indications, such as post-exposure prophylaxis (PEP), chronic hepatitis B management, or treatment for HIV and other opportunistic infections. Records missing key data to assess PrEP indications were excluded from the analysis. We determined total PrEP usage with TDF/FTC,TAF/FTC, and cabotegravir, and did not separate data for each product due to similar levels of HIV protection provided.^17^ IQVIA data do not identify individuals, but do include data on sex, state and county of US residence, and age. A subset of records also has data on race and ethnicity, which are determined by IQVIA using probabilistic matching of prescription data with data from consumer credit reporting systems.

### Trends in rates of PrEP use by race, ethnicity and sex

To describe trends in PrEP utilization over time, we tabulated rates per 100,000 population overall, by race and ethnicity, by sex, by region, and by race and ethnicity and sex within region.^8^ Because race and ethnicity data were missing for nearly two thirds of the PrEP users, we assumed that the proportional distribution of race and ethnicity among PrEP users was the same for prescriptions missing race and ethnicity as for those with matched race and ethnicity data and conducted a sensitivity analysis. We reported race and ethnicity-specific rates of PrEP use for each annual era of PrEP use. The denominators used to calculate PrEP rates and PnRs can be found in Supplemental Tables S1 and S2

### Trends in race and ethnicity- and sex-specific PrEP-to-need ratios and PrEP equity ratios

We calculated race and ethnicity- and sex-specific PnRs for the US overall and within each US region from 2012 to 2021. PnRs use HIV new diagnoses as the denominator; new diagnoses in this setting serve as a subpopulation-specific measure of risk of contracting HIV (a proportionate proxy of the number of people at risk for HIV infection). We defined equitable PrEP use as equal race and ethnicity-, sex-, and year-specific PnRs within a region. We defined the PER as the ratio of two PnRs for a given potential disparity assessment, including race and ethnicity- (e.g., Black-to-White, Hispanic-to-White) and sex-specific PnRs (Female-to-Male). For this metric, equitable PrEP use is defined as a PER of 1.0, and smaller PERs represent larger inequities in PrEP use for the disparity population (e.g., the numerator population).

### Sensitivity analysis

To address potential biases inherent to the assumption that PrEP prescriptions missing race and ethnicity data had the same distribution as those with nonmissing race and ethnicity data, we conducted sensitivity analyses to assess the impact of race redistribution on PrEP use metrics. Our deterministic method applied an alternative redistribution algorithm of PrEP users with missing race based on the findings of a published imputation study from population-based data. The prior study indicated that unreported race data is attributable disproportionately to Black and Hispanic people.^18^ We used the estimated rates of race-specific increases from before and after imputation from the previous study and calibrated these rates of increase to the observed race distribution of our data. We report the PnRs and PERs from the sensitivity analyses to provide context to how sensitive our results are to our assumptions around missing race.

The analyses were performed using SAS version 9.4 (SAS Institute Inc).

### Role of the funding source

Access to data was provided in kind by Gilead Sciences. Gilead Sciences had no role in the analysis or interpretation of data, or in the writing of the manuscript. The funders had no role in the design, data collection analysis, interpretation, or writing of this report.

## Results

Overall, the number of PrEP users increased from 9626 in 2012 to 363,957 in 2021 (Table 1). Across all race and ethnicity groups, sex, and regions, rates of PrEP use increased throughout the period. By 2021, the rate of PrEP use per population size was similar in Black non-Hispanic (129/100,000) and White non-Hispanic (122/100,000) populations but slightly lower among Hispanic (95/100,000) populations. In 2021, the male-specific rate per population (241/100,000) was higher than the female-specific rate (20/100,000) and the rate of PrEP use was highest in the Northeast (163/100,000) and lowest in the Midwest (95/100,000).

**Table 1:** Number and rates of annual pre-exposure prophylaxis (PrEP) users, overall, and by race and ethnicity, sex, and region, United States 2012–2021.

	n (rate, per 100,000 population)									
	2012	2013	2014	2015	2016	2017	2018	2019	2020	2021
Overall	9626 (4)	10,385 (4)	23,094 (9)	56,462 (21)	103,862 (38)	151,745 (55)	214,639 (77)	266,484 (96)	295,416 (106)	363,957 (129)
**Race & Ethnicity**										
Black, non- Hispanic	1862 (5)	1580 (4)	2823 (7)	6735 (17)	12,864 (32)	19,274 (48)	28,025 (70)	35,332 (87)	41,154 (101)	50,671 (129)
Hispanic	1297 (2)	1506 (3)	3052 (5)	7660 (13)	14,747 (24)	22,778 (37)	34,070 (54)	42,809 (67)	49,383 (78)	62,663 (95)
White, non-Hispanic	5454 (3)	6305 (3)	15,639 (8)	39,153 (20)	71,254 (36)	102,450 (52)	142,557 (72)	176,276 (90)	192,119 (98)	234,437 (122)
**Sex**										
Male	5887 (5)	6779 (5)	19,379 (15)	51,190 (39)	95,130 (71)	140,028 (104)	198,206 (146)	245,547 (180)	271,658 (199)	334,804 (241)
Female	3695 (3)	3570 (3)	3669 (3)	5210 (4)	8642 (6)	11,630 (8)	16,314 (12)	20,812 (15)	23,661 (17)	28,870 (20)
**Region**										
Midwest	1321 (2)	1492 (3)	3680 (7)	8921 (16)	16,748 (29)	24,550 (43)	34,341 (60)	42,470 (74)	44,462 (77)	54,455 (95)
Northeast	2096 (4)	2641 (6)	5702 (12)	14,557 (30)	27,167 (57)	40,278 (84)	54,797 (114)	65,336 (137)	66,210 (138)	77,965 (163)
South	3785 (4)	3305 (3)	6650 (7)	16,007 (16)	30,059 (30)	44,505 (43)	67,843 (65)	89,467 (85)	110,845 (105)	140,926 (134)
West	2374 (4)	2879 (5)	7001 (11)	16,918 (27)	29,794 (47)	42,283 (65)	57,469 (88)	68,958 (105)	73,599 (112)	90,224 (137)

**Fig. 1:**
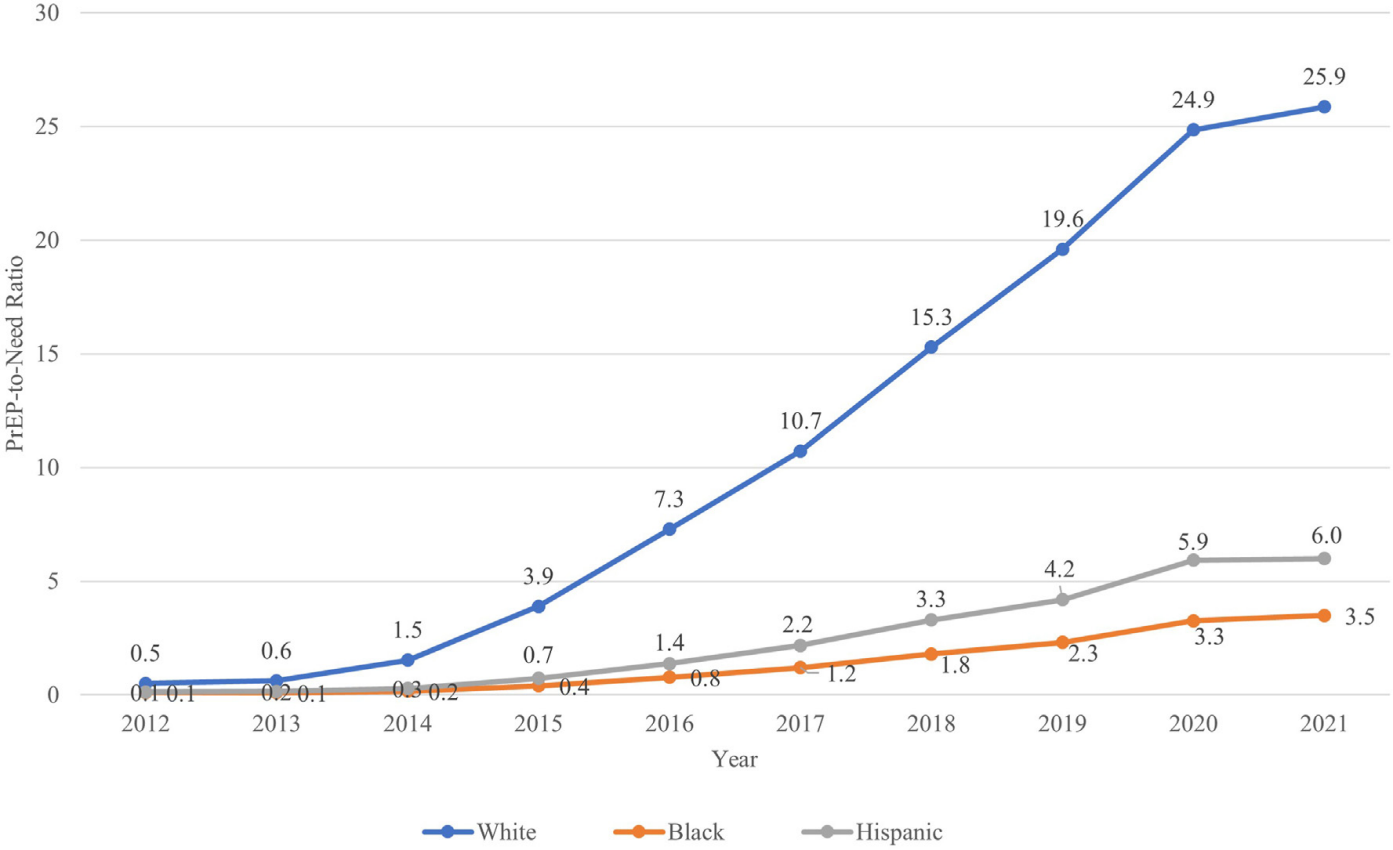
**PrEP-to-need ratio by race and ethnicity, United States, 2012–2021**.

**Fig. 2:**
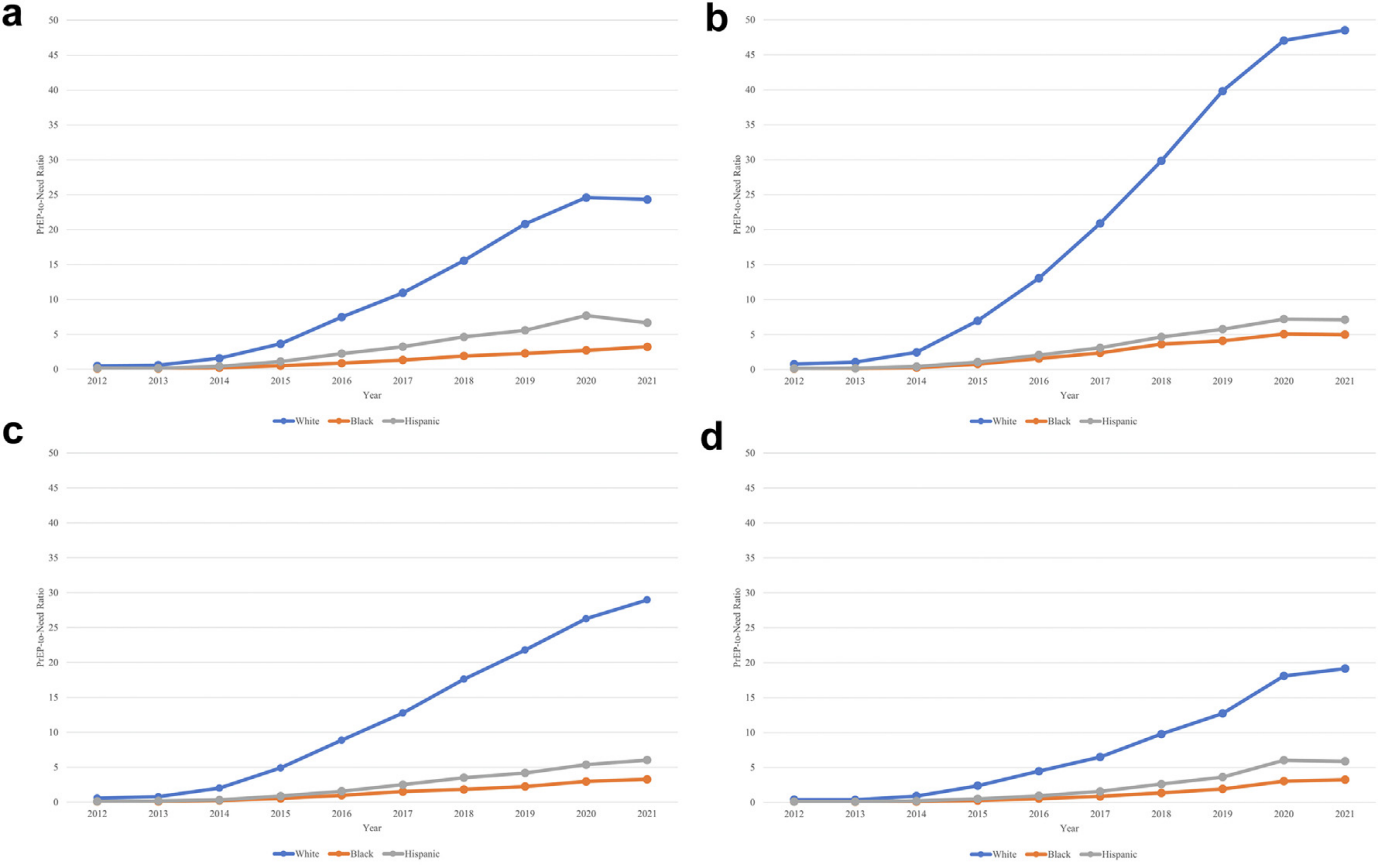
**PrEP-to-Need Ratio by Raceand Ethnicity, within Region, United States, 2012–2021**. The 4 regions are shown as panels: (a) Midwest, (b) Northeast, (c) West, (d) South. ∗Note: Washington DC is included in the South.

Considering race and ethnicity-specific trends in PrEP provision relative to the need in the population (PnR), several patterns were observed. First, in every region and in every year, the PnR was highest for White persons, intermediate for Hispanic persons, and lowest for Black persons (Fig. 1, Fig. 2). Second, across years, overall, the PnRs were consistently highest in the Northeast, and lowest in the South. Third, race and ethnicity was a more important factor than region in determining levels of PrEP use relative to need: for example, White residents in the South (the region with the lowest overall PnR) had a higher PnR than Black residents in the Northeast (the region with the highest overall PnR) (Fig. 2; Supplemental Table S3).

**Fig. 3:**
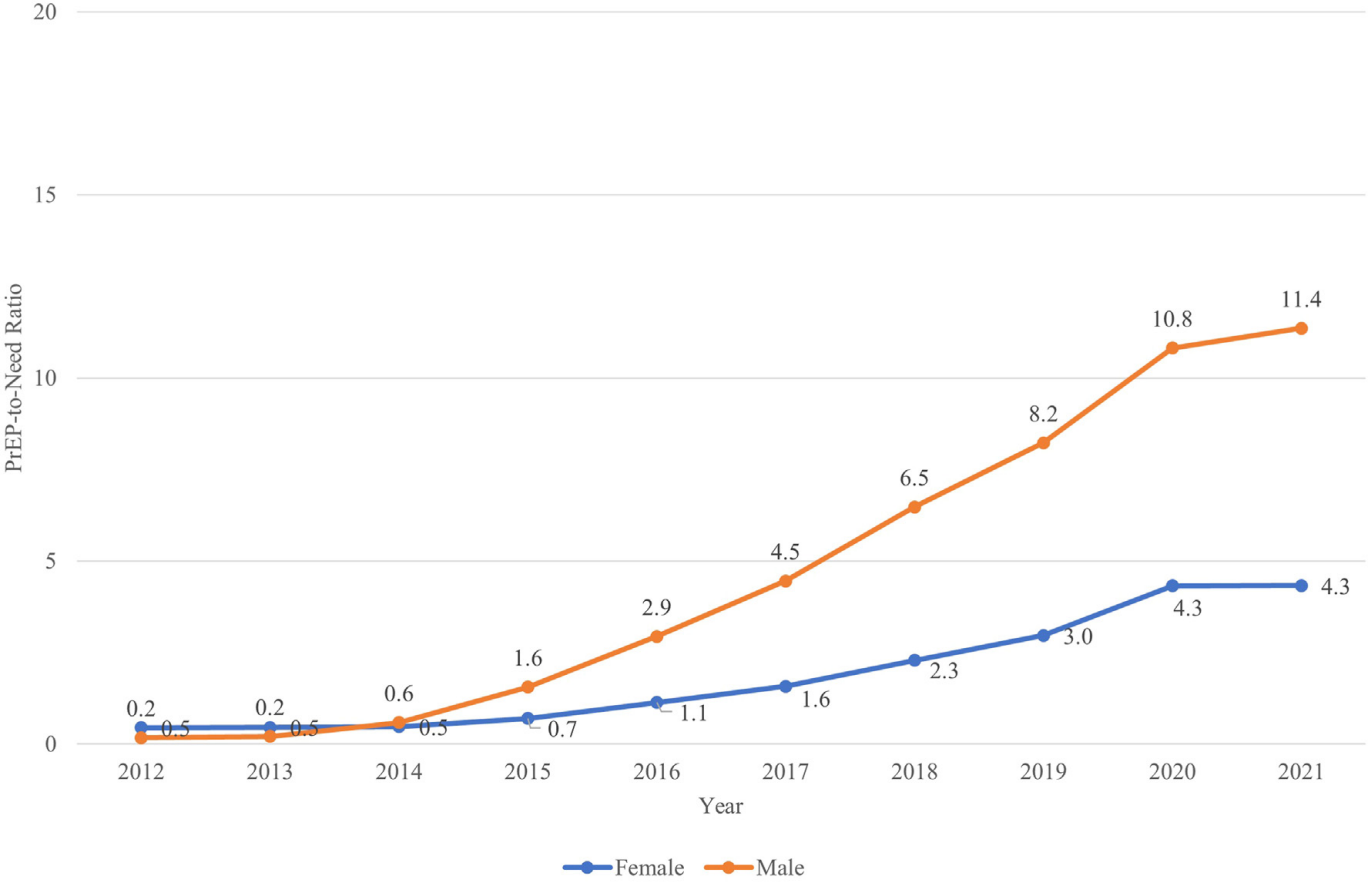
**PrEP-to-need ratio by sex, United States, 2012**–**2021**.

**Fig. 4:**
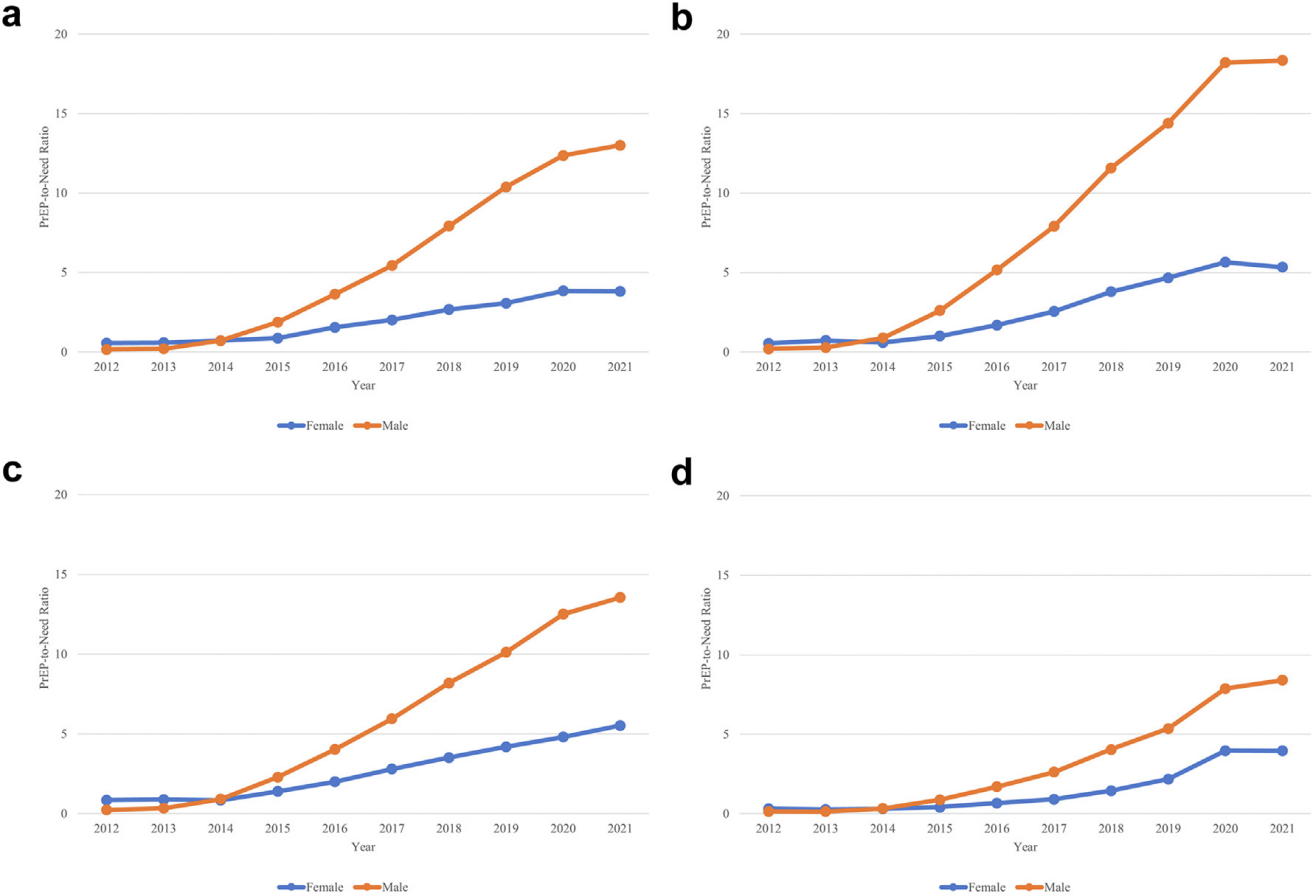
**PrEP-to-Need Ratio by Sex, within Region, United States, 2012–2021**. The 4 regions are shown as panels: (a) Midwest, (b) Northeast, (c) West, (d) South. ∗Note: Washington DC is included in the South.

Considering sex-specific trends in PrEP use relative to population need, the PnR was higher for males than females overall and within region (Fig. 3, Fig. 4). The PnRs by sex were similar at the start of PrEP but a gap between PnR values was observed in 2015 and has continued to widen over time.

**Fig. 5:**
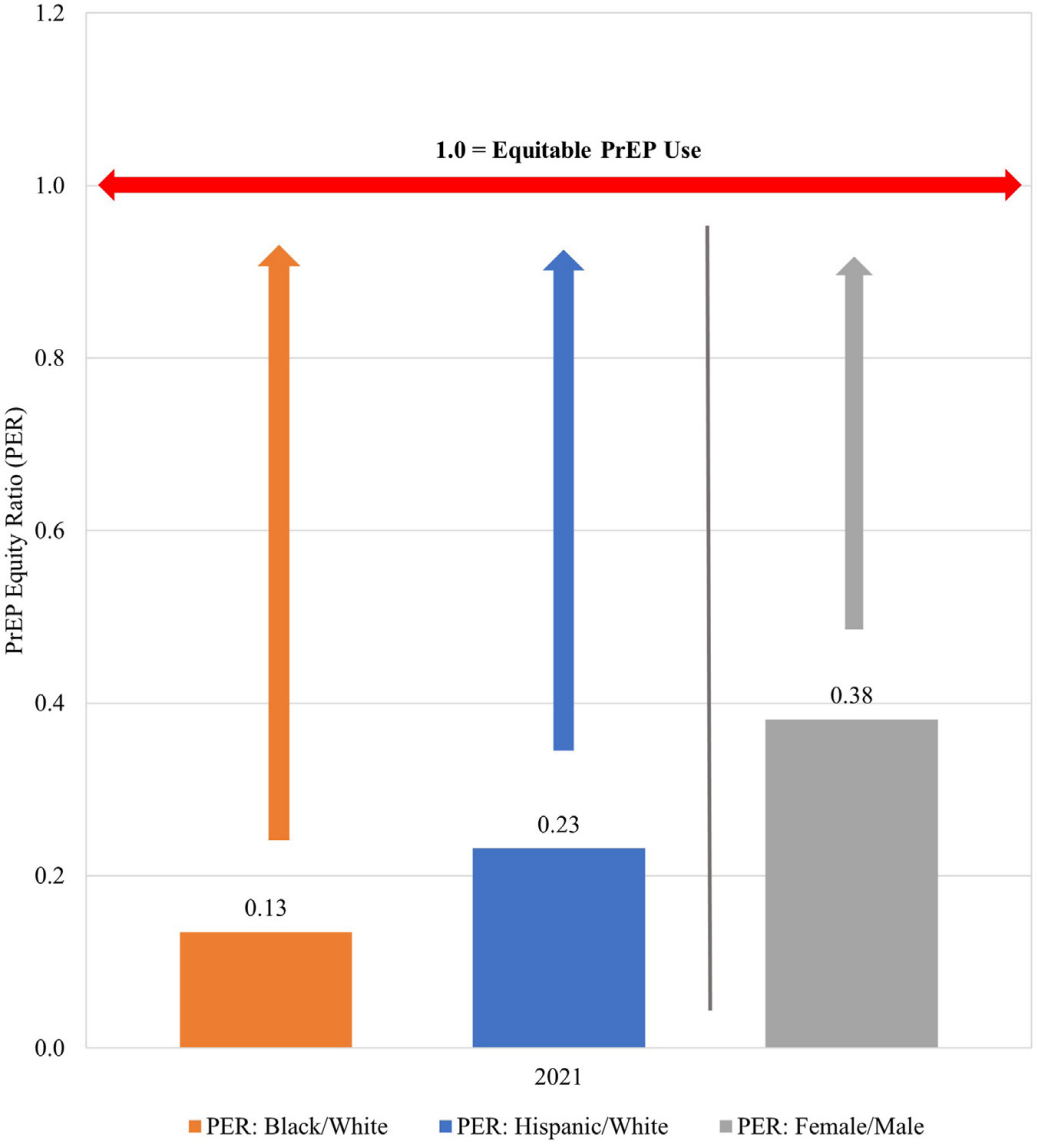
**PrEP Equity Ratio by Race and Ethnicity and Sex, United States, 2021**. Length of arrows represent the extent of inequities compared to equitable use by race, ethnicity, or sex.

**Fig. 6:**
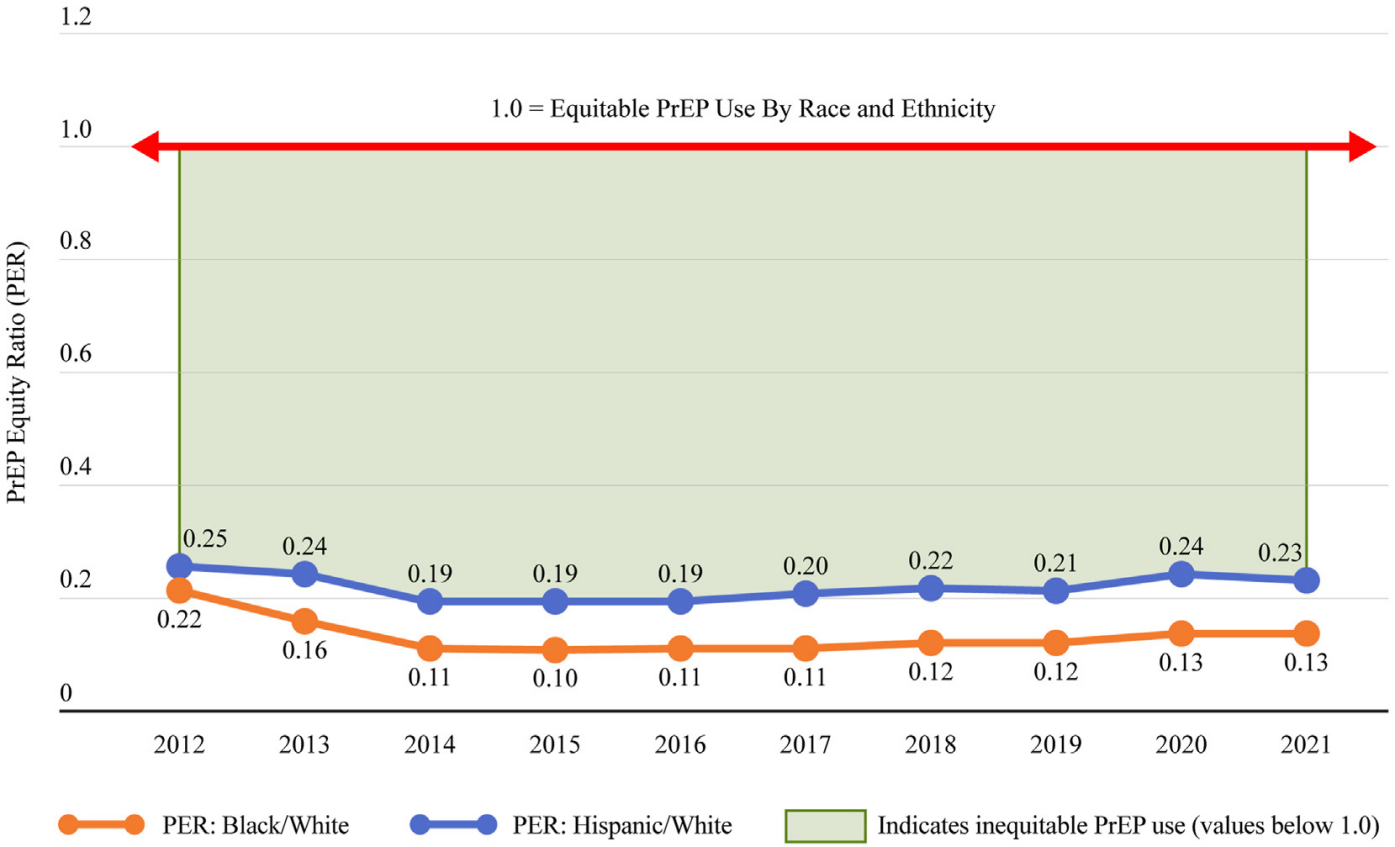
**Trend in black/white and hispanic/white PrEP equity ratio, United States, 2012–2021**.

**Fig. 7:**
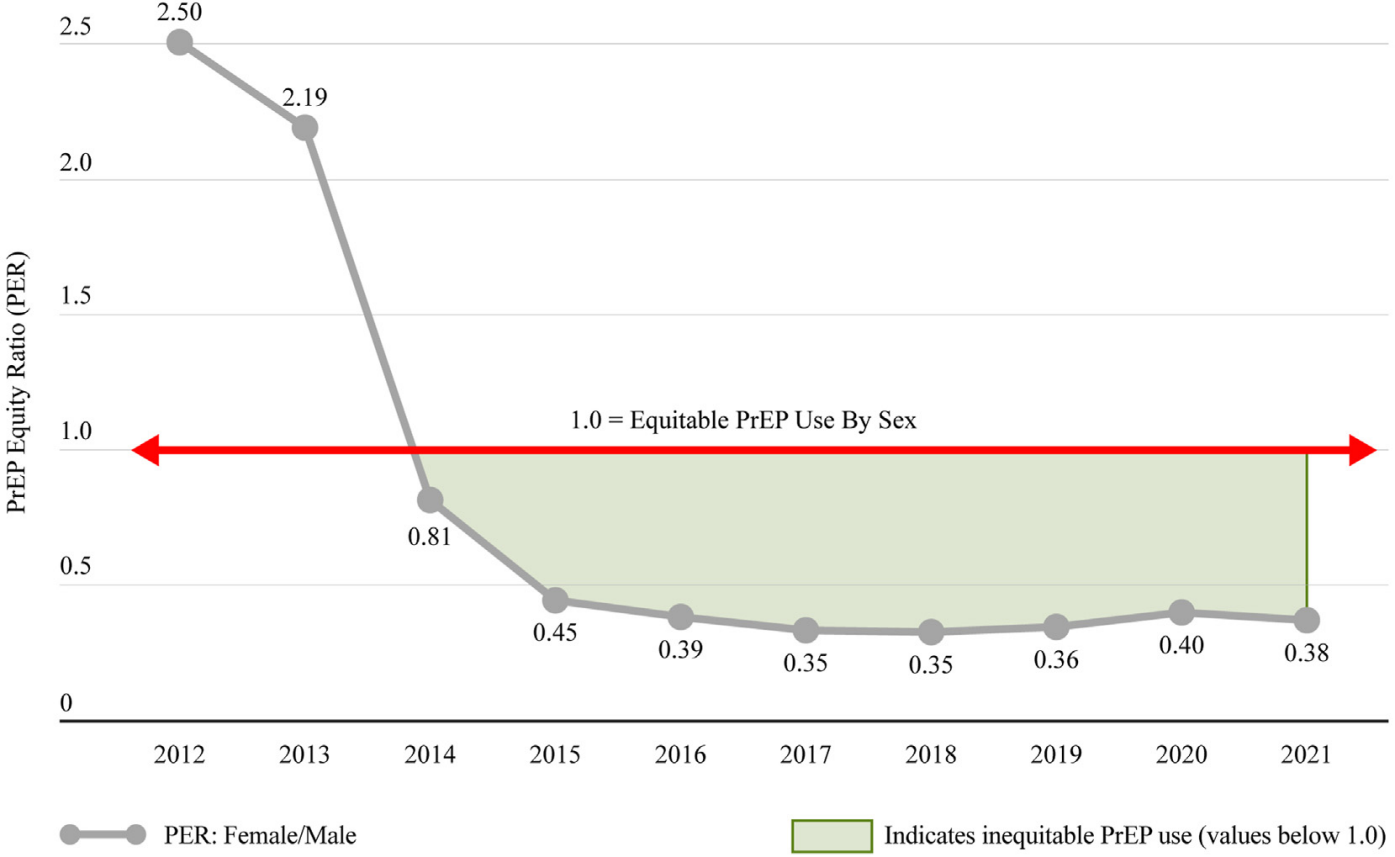
**Trend in female/male PrEP equity ratio, United States, 2012–2021**.

In 2021, the PER for Black-to-White, Hispanic-to-White and female-to-male were well below an equitable ratio of 1.0 (Fig. 5). With respect to the PER over time, we observed that the PER (e.g., the gap between Black or Hispanic and White PnR values) grew early after the start of PrEP and persisted at a level much below equitable PrEP use throughout the study period (Fig. 6). Similarly, there was a gap between female and male PnR values, indicating inequitable PrEP use among women relative to their epidemic need (Fig. 7).

The sensitivity analysis to assess the effect of bias attributable to our base case (naïve) assumption that the distribution of missing race was the same as the distribution of reported race documents a small amount of bias towards the null with respect to PrEP Equity Ratio results. Year- and race/ethnicity-specific bias-adjusted PrEP equity ratio results were higher (i.e., closer to the null value of 1.0) than the naïve PERs by an average of 10% (range: 6%–16%; Supplemental Table S4), but did not result in a change in direction of effect or a meaningful difference in the extent of the inequities.

## Discussion

The US HIV epidemic is marked by disparities by sex, race, ethnicity, and geography. In evaluating the implementation of a prevention technology, such as PrEP, it is important to assess both progress towards increasing coverage, and the extent to which that progress is equitable. Traditional metrics for assessing coverage of programs, such as proportions of eligible people who receive services^19^ or coverage rates per 100,000 population, are essentially equality-based metrics – they describe the extent to which all groups (race and ethnicity, region, age, sex) have equivalent use of a health service or technology. Because the *risk* of HIV infection is higher among Black people than for people of other races and ethnicities (i.e., Black people accounted for 40% of new HIV diagnoses in the US 2021, but only 13% of the US population), an equitable outcome should be that Black people are equally represented among PrEP users in proportion to their epidemic burden. According to our data, the reverse is true: Black and Hispanic populations, who experience disproportionate shares of HIV infections, have lower PrEP provision relative to population need than do White populations. We advance the use of the PrEP Equity Ratio as a metric to quantify these relationships and to offer targets for monitoring whether interventions and programs to increase PrEP uptake actually impact equitable use.

Other approaches have been used to assess equitable PrEP use. For example, data^20^ from the CDC National HIV Behavioral Surveillance System^21^^,^^22^ have been used to assess the proportion of PrEP-eligible men who were prescribed PrEP. If PrEP eligibility criteria had equal sensitivity in identifying people with elevated risk of acquiring HIV, PrEP use with a denominator of eligible men should account for differences in risk of acquiring between men of different race/ethnicities. However, the risk of HIV infection for Black MSM is higher than the risk of White MSM even after controlling for other behaviors,^23^ and historical PrEP eligibility criteria might have been under sensitive in identifying Black MSM who might benefit from PrEP.^24^ This is likely because Black MSM are more likely to have Black partners (who have a higher prevalence of HIV) and to have partners with unsuppressed viral load.^23^ Therefore, comparing proportions of MSM with PrEP indications based on guidelines that don't consider risks of infection might underestimate gaps in need. Using population incidence denominators for an equity metric might still have residual bias if the likelihood of being diagnosed and reported to state surveillance programs differs by race; unfortunately, there is evidence that this might be the case.^25^ Inequities in geospatial access to PrEP have been reported, overall in the United States,^26^ in rural areas,^27^ and within cities.^28^

To make recommendations for how to decrease PrEP inequities, it is important to recognise the barriers to PrEP uptake for Black and Hispanic people. For example, a major driver of lack of interest in PrEP among a nationally representative sample of US Black people in 2016 was low perceived risk for HIV infection; less contact with healthcare providers was also associated with lower willingness to use PrEP.^29^ Racial segregation within cities and physical distance from PrEP service providers also likely shape lower access to PrEP services.^30^ For some Black and Hispanic people, individual level (e.g., knowledge, attitudes, beliefs, concern about side effects, competing priorities for food or shelter) and network-level (e.g., provider unconscious bias, racism, sexism, homophobia, transphobia, stigmatization) barriers have been summarized, and some potentially effective interventions have been described.^31^ Black and Hispanic Americans are also less likely to have health insurance,^32^ and Southern states, where a disproportionate number of Black and Hispanic people live, are less likely to have Medicaid expansion and/or PrEP drug assistance programs – both of which have been associated with higher PrEP use.^23^^,^^33^ For women, stigma, costs, and lack of awareness of the potential risks of male partners have been historical barriers.^34^^,^^35^ Across all of these categories, it is critical to work towards developing estimates for people with intersectional risks – for example, estimates for men in the South, estimates for women by race/ethnicity, and estimates for MSM by age and race. Because of missingness of some stratifying variables in the pharmaceutical data (especially race/ethnicity), the availability of these intersectional estimates is currently limited.

The argument to prioritise equitable PrEP programs is sufficiently predicated on social justice alone, but equitable PrEP programs will also have substantially larger impact on achieving public health goals for ending the HIV epidemic than inequitable programs. Goedel et al. simulated disparities in HIV incidence between Black and White MSM across a range of equitable and inequitable PrEP coverage scenarios, and found that PrEP inequities (PrEP equity ratios, in the parlance of this manuscript) in the 7–10 fold range (e.g., relative PnR of 7–10 for White MSM and 1 for Black MSM) would lead to increased disparities in HIV incidence.^36^ Note that, because Black MSM in the US are estimated to have HIV incidence rates that are over 10 times the incidence rates of White MSM,^37^ having *equal* proportions of Black and White MSM using PrEP would, in Goedel's scenarios, still be predicted to exacerbate existing Black/White disparities in incidence. It is concerning that after Goedel's observation, the PERs for both Black (2021 PER = 0.13) and Hispanic (2021 PER = 0.23) people dropped to the range in which we expect incidence disparities to grow.

Our data are subject to important limitations. First, data on race and ethnicity for PrEP users was available only for about 34% of PrEP prescriptions. It is likely that missingness might be differential by race and ethnicity, because commercial credit records are sourced from credit card usage and payment patterns, and Black Americans are less likely than other Americans to hold a credit card.^38^ However, to account for this, we performed a sensitivity analysis with an alternate redistribution of PrEP use by race and although the results showed a slight narrowing of the PrEP-to-Need ratio gap between racial groups, it did not change our overall findings. We are aware of the need to improve methods to describe more fully the race of persons using PrEP in commercial pharmacy data and are exploring methods for this. Second, our estimates of PrEP utilization are minimum estimates because closed healthcare systems (e.g., Veteran's Administration, HMOs with their own pharmacies) are not represented in the commercial pharmacy data. Although exact numbers are not available, we estimate that those with TRICARE and CHAMPVA coverage represent 3.5% of US people in care. Kaiser Permanente, a major healthcare provider not included in IQVIA data, reports having 12.6 million members in 2022 – about 3.8% of the US population. Thus, our best estimate is that about 7% of the population in care through these care systems would not be represented in our PrEP use data. It is not clear how the patterns of PrEP use by race, ethnicity and sex in those settings might alter our results, if available. Third, based on our previous validation studies, we used 87% “upweighting” for unclassified prescriptions,^8^ but in this and other contemporary analyses, we now use 94% because a greater proportion of prescriptions are likely for PrEP as the overall number of PrEP users increases. Because this upweighting is not differential by race and ethnicity, it should not have important impacts on ratio-based measures. Fourth, commercial prescription datasets include data on sex, but not gender. Transgender and non-binary people experience high risks for HIV infection,^39^ many have PrEP indications,^40^ and it is important to continue to explore approaches to represent their PrEP experiences and equity measures as data allow. Finally, we acknowledge that the PER measure is a “ratio of ratios”; the statistical value of these types of indicators has been debated.^41^ However, the primary concern about these measures relates to the calculation of confidence intervals. Here, we use the PER as an indicator of relative inequity, but do not attempt to put confidence intervals around these measures. Our numerator and denominator data for PnR are both population-based, so the external validity of our results should be good. It is possible that PnR values for 2020 could be biased upwards, because there were lower numbers of people diagnosed with HIV during 2020; this was likely attributable to the COVID pandemic.^42^

It is possible that alignment between PrEP prescriptions and individual risk is not perfect, such that some PrEP might be prescribed to people with minimal risks for infection.^43^ Such PrEP prescriptions are unlikely to contribute to reductions in HIV incidence. Further, it is possible that the frequency of such “futile” PrEP prescriptions might vary by important demographic and risk subgroups. Clearly, the prevention impact of a single PrEP prescription varies by the risk of the person to whom PrEP is prescribed.^36^

In the United States, disparities by race and ethnicity in PrEP uptake relative to epidemic need (i.e., PrEP inequities) have existed since the initial regulatory approval of PrEP and have continued to grow over the first decade of PrEP availability in the US. If we are not providing PrEP equitably, then we are also not getting the most prevention value from the PrEP that is being prescribed. To help monitor these inequities, we recommend including race and ethnicity- and sex-specific PnRs and the PrEP Equity Ratio as monitoring tools at the national, regional and state levels. These PnR data are already available in tabular form and at national, regional and state levels, and for most EHE counties on AIDSVu.org,^44^ and should also be made available through other national^19^ and local data portals and used by community planning bodies. Our prior work has documented that Medicaid expansion and PrEP Drug Assistance Programs are associated with higher PnRs at the state level^33^; expansion of such programs might help to achieve EHE goals. Other structures that minimize financial and other barriers to accessing PrEP should also be prioritized.^45^^,^^46^ We recommend new Public Health Service guidance liberalizing PrEP eligibility criteria^47^ be put into practice, especially because the guidance offers the potential to liberalize offering of PrEP to women if implemented systematically. In light of differences in the prevalence of HIV in partner pools by race and ethnicity, it is critical to implement the new CDC guidance so that anyone who feels they would benefit from PrEP will be considered as a PrEP candidate.^47^ Fourth, although it is a long process, we must continue to prioritize developing, testing and disseminating effective stigma reduction interventions addressing intersectional stigmas around sex, race and ethnicity, and gender minority.^31^^,^^46^^,^^48^

We must reframe our programs and monitoring metrics to push beyond PrEP equality and towards PrEP equity. Ultimately, PrEP equity is good public health; we will avert the most new HIV infections when we get PrEP to the people who need it most.^49^

## Contributors

**PS:** conceptualisation, formal analysis, funding acquisition, investigation, methodology, project administration, resources, visualisation, writing – original draft. PS had access to the raw data and final responsibility to submit for publication.

**SD:** data curation, methodology, project administration, supervision, data verification, writing – original draft.

**AC:** conceptualisation, methodology, writing – review & editing.

**KH:** conceptualisation, methodology, writing – review & editing. **MJ:** data curation, formal analysis, data verification, methodology, writing – review & editing.

**JG:** methodology, writing – review & editing.

**GL:** data curation, formal analysis, data verification; writing – review & editing.

**SW:** data curation, project administration, writing – review & editing.

**AJS:** conceptualisation, data curation, formal analysis, methodology, resources, supervision, validation, writing – original draft.

## Data sharing statement

All data on PrEP use and HIV diagnoses used in these analyses are freely available for download and analysis at https://aidsvu.org/resources/#/datasets. Data on HIV diagnoses are also available at CDC's NCHHSTP AtlasPlus at https://www.cdc.gov/nchhstp/atlas/index.htm.

## CDC Disclaimer

The findings and conclusions in this report are those of the authors and do not necessarily represent the official position of the Centers for Disease Control and Prevention.

## Declaration of interests

Staff funding for Sullivan was provided, in part, by the Center for AIDS Research at Emory University (P30 AI050409). Sullivan, DuBose, Juhasz and Le are supported by a grant from Gilead Sciences to Emory University.
