# Supplementary material for: Equity of PrEP uptake by race, ethnicity, sex and region in the United States in the first decade of PrEP: a population-based analysis
Source: Lancet Reg Health Am. 2024 Apr 22;33:100738. doi: 10.1016/j.lana.2024.100738 (PMC11041841; doi:10.1016/j.lana.2024.100738)
Supplement: Supplementary Tables [file mmc1.docx]

**Supplementary Material**

**Supplemental Table 1. Population Sizes, Overall, and by Race and Ethnicity, Sex, and Region, United States 2012-2021**

|  | **2012** | **2013** | **2014** | **2015** | **2016** | **2017** | **2018** | **2019** | **2020*** | **2021** |
| --- | --- | --- | --- | --- | --- | --- | --- | --- | --- | --- |
| **Overall  (ages 13+)** | 264181732 | 266325382 | 269059751 | 271596787 | 273260094 | 276044000 | 277672731 | 279100758 | 279100758 | 283032473 |
|  |  |  |  |  |  |  |  |  |  |  |
| **Race & Ethnicity (all ages)**** |  |  |  |  |  |  |  |  |  |  |
| Black, non-  Hispanic | 38466763 | 38810193 | 39270537 | 39600891 | 39719644 | 40132047 | 40310242 | 40599428 | 40599428 | 39273516 |
| Hispanic | 56592425 | 57568600 | 58793731 | 59933510 | 60764560 | 62152594 | 62917648 | 63616505 | 63616505 | 65768124 |
| White, non-Hispanic | 197266965 | 197414474 | 197433062 | 197560927 | 197515618 | 197308581 | 197064732 | 196832007 | 196832007 | 192768696 |
|  |  |  |  |  |  |  |  |  |  |  |
| **Sex  (ages 13+)** |  |  |  |  |  |  |  |  |  |  |
| Male | 128888888 | 130053130 | 131301422 | 132674523 | 133482308 | 134884955 | 135687949 | 136347041 | 136347041 | 139183936 |
| Female | 135207426 | 136293014 | 137744845 | 138984453 | 139838942 | 141159043 | 141984787 | 142753721 | 142753721 | 143848534 |
|  |  |  |  |  |  |  |  |  |  |  |
| **Region  (ages 13+)** |  |  |  |  |  |  |  |  |  |  |
| Midwest | 55977349 | 56301935 | 56558460 | 56766479 | 56851220 | 57125473 | 57323481 | 57425918 | 57425918 | 57961503 |
| Northeast | 47208679 | 47440547 | 47694857 | 47803944 | 47846597 | 48157153 | 47898075 | 47848606 | 47848606 | 48910574 |
| South | 97172717 | 98170515 | 99571827 | 100861688 | 101856591 | 103279181 | 104412910 | 105294482 | 105294482 | 106970872 |
| West | 60747037 | 61362698 | 62217050 | 63193556 | 63762410 | 64587804 | 65245006 | 65721158 | 65721158 | 66285843 |
| Population data source: U.S. Census Bureau - ACS 1-Year Estimates | | | | |  |  |  |  |  |  |
| *2019 population data used to calculate 2020 PrEP rates due to lack of 2020 population data  **New diagnoses data is available for ages 13 and older, while population data (used to calculate PrEP rates) includes all ages | | | | | | | |  |  |  |

**Supplemental Table 2. New HIV Diagnoses, Overall, and by Race and Ethnicity, Sex, and Region, United States 2012-2021**

|  | **2012** | **2013** | **2014** | **2015** | **2016** | **2017** | **2018** | **2019** | **2020** | **2021** |
| --- | --- | --- | --- | --- | --- | --- | --- | --- | --- | --- |
| **Overall (ages 13+)** | 41,099 | 39,713 | 40,470 | 40,285 | 39,962 | 38,775 | 37,736 | 36,817 | 30,575 | 36,126 |
|  |  |  |  |  |  |  |  |  |  |  |
| **Race & Ethnicity (ages 13+)** |  |  |  |  |  |  |  |  |  |  |
| Black, non-  Hispanic | 17,241 | 16,497 | 16,812 | 16,749 | 16,598 | 16,107 | 15,594 | 15,296 | 12,643 | 14,522 |
| Hispanic | 10,070 | 9,864 | 10,365 | 10,479 | 10,699 | 10,438 | 10,353 | 10,243 | 8,337 | 10,465 |
| White, non-Hispanic | 10,609 | 10,234 | 10,207 | 10,062 | 9,773 | 9,547 | 9,326 | 8,989 | 7,732 | 9,062 |
|  |  |  |  |  |  |  |  |  |  |  |
| **Sex (ages 13+)** |  |  |  |  |  |  |  |  |  |  |
| Male | 32,842 | 32,000 | 32,800 | 32,827 | 32,397 | 31,424 | 30,601 | 29,819 | 25,102 | 29,461 |
| Female | 8,257 | 7,713 | 7,670 | 7,458 | 7,565 | 7,351 | 7,135 | 6,998 | 5,473 | 6,665 |
|  |  |  |  |  |  |  |  |  |  |  |
| **Region (ages 13+)** |  |  |  |  |  |  |  |  |  |  |
| Midwest | 5,356 | 5,250 | 5,080 | 5,213 | 5,145 | 5,099 | 4,941 | 4,767 | 4,106 | 4,815 |
| Northeast | 7,381 | 6,858 | 6,888 | 6,431 | 6,199 | 5,988 | 5,552 | 5,317 | 4,241 | 4,988 |
| South | 20,072 | 19,764 | 20,116 | 20,345 | 20,190 | 19,645 | 19,225 | 18,950 | 15,537 | 18,703 |
| West | 7,559 | 7,196 | 7,764 | 7,732 | 7,890 | 7,596 | 7,581 | 7,387 | 6,391 | 7,210 |

**Supplemental Table 3. Number and Rates of Annual Pre-exposure Prophylaxis (PrEP) Users, by Race and Ethnicity and Sex, within Region, United States 2012–2021**

|  | n (rate, per 100,000 population) | | | | | | | | | |
| --- | --- | --- | --- | --- | --- | --- | --- | --- | --- | --- |
|  | **2012** | **2013** | **2014** | **2015** | **2016** | **2017** | **2018** | **2019** | **2020** | **2021** |
| **Midwest** |  |  |  |  |  |  |  |  |  |  |
| Black, non-Hispanic | 212 (3) | 202 (3) | 477 (7) | 1138 (16) | 2088 (30) | 3106 (44) | 4388 (63) | 5070 (72) | 5248 (75) | 6717 (95) |
| Hispanic | 110 (2) | 102 (2) | 286 (6) | 724 (14) | 1477 (28) | 2162 (40) | 3095 (57) | 3871 (70) | 3997 (72) | 4831 (87) |
| White, non-Hispanic | 852 (2) | 1022 (2) | 2677 (5) | 6684 (13) | 12645 (24) | 18553 (36) | 25879 (50) | 32322 (63) | 33956 (66) | 41346 (81) |
|  |  |  |  |  |  |  |  |  |  |  |
| Male | 768 (3) | 926 (3) | 3062 (11) | 8064 (29) | 15241 (55) | 22598 (81) | 31808 (113) | 39408 (140) | 41594 (148) | 51017 (181) |
| Female | 545 (2) | 555 (2) | 615 (2) | 836 (3) | 1475 (5) | 1920 (7) | 2482 (8) | 2979 (10) | 2842 (10) | 3400 (12) |
|  |  |  |  |  |  |  |  |  |  |  |
| **Northeast** |  |  |  |  |  |  |  |  |  |  |
| Black, non-Hispanic | 282 (5) | 412 (7) | 675 (11) | 1937 (31) | 3672 (59) | 5556 (88) | 7717 (124) | 8708 (140) | 8494 (137) | 9735 (156) |
| Hispanic | 306 (4) | 357 (5) | 851 (11) | 2064 (26) | 3979 (50) | 5921 (72) | 8305 (101) | 9842 (118) | 9764 (117) | 11539 (139) |
| White, non-Hispanic | 1289 (3) | 1665 (4) | 3822 (10) | 9857 (27) | 18185 (49) | 26872 (73) | 36107 (99) | 43685 (121) | 44825 (124) | 53028 (147) |
|  |  |  |  |  |  |  |  |  |  |  |
| Male | 1121 (5) | 1539 (7) | 4771 (21) | 13133 (57) | 24784 (107) | 36879 (158) | 50165 (217) | 59939 (259) | 61231 (265) | 72329 (312) |
| Female | 972 (4) | 1098 (4) | 924 (4) | 1415 (6) | 2369 (10) | 3398 (14) | 4623 (19) | 5395 (22) | 4962 (20) | 5586 (23) |
|  |  |  |  |  |  |  |  |  |  |  |
| **South** |  |  |  |  |  |  |  |  |  |  |
| Black, non-Hispanic | 1238 (6) | 861 (4) | 1382 (6) | 2976 (13) | 5773 (25) | 8711 (37) | 13536 (57) | 18742 (79) | 24256 (102) | 30167 (127) |
| Hispanic | 466 (2) | 472 (2) | 848 (4) | 2130 (10) | 4058 (19) | 6633 (30) | 11081 (49) | 15147 (66) | 20304 (88) | 26025 (113) |
| White, non-Hispanic | 1760 (3) | 1705 (2) | 4058 (6) | 10372 (15) | 19288 (27) | 27770 (40) | 41206 (59) | 53061 (75) | 63268 (90) | 80781 (115) |
|  |  |  |  |  |  |  |  |  |  |  |
| Male | 2352 (5) | 2124 (4) | 5314 (11) | 14250 (29) | 27249 (55) | 40784 (81) | 62110 (122) | 81140 (159) | 98948 (194) | 126137 (247) |
| Female | 1407 (3) | 1160 (2) | 1311 (3) | 1737 (3) | 2773 (5) | 3700 (7) | 5705 (11) | 8305 (15) | 11856 (22) | 14664 (27) |
|  |  |  |  |  |  |  |  |  |  |  |
| **West** |  |  |  |  |  |  |  |  |  |  |
| Black, non-Hispanic | 131 (4) | 105 (3) | 290 (9) | 685 (20) | 1331 (39) | 1900 (56) | 2383 (68) | 2812 (80) | 3156 (90) | 4053 (115) |
| Hispanic | 365 (2) | 506 (2) | 1006 (5) | 2683 (12) | 5139 (23) | 7934 (34) | 11426 (49) | 13695 (58) | 15020 (64) | 19880 (84) |
| White, non-Hispanic | 1553 (4) | 1913 (5) | 5082 (13) | 12241 (32) | 21136 (55) | 29254 (76) | 39339 (101) | 47208 (122) | 50069 (129) | 59281 (153) |
|  |  |  |  |  |  |  |  |  |  |  |
| Male | 1607 (5) | 2146 (7) | 6196 (20) | 15692 (50) | 27779 (88) | 39657 (124) | 53964 (167) | 64836 (199) | 69617 (213) | 84965 (260) |
| Female | 759 (2) | 734 (2) | 796 (3) | 1216 (4) | 2008 (6) | 2594 (8) | 3478 (11) | 4107 (12) | 3971 (12) | 5188 (16) |

**Supplemental Table 4. Prep-to-Need Ratios (PnR) and Prep Equity Ratios (PER) for Base Case versus Sensitivity Analyses**

|  | **Base Case PnR** | | | **Sensitivity Analyses PnR** | | | **Base Case PER** | | **Sensitivity Analyses PER** | |
| --- | --- | --- | --- | --- | --- | --- | --- | --- | --- | --- |
| **Year** | **White** | **Black** | **Hispanic** | **White** | **Black** | **Hispanic** | **Black:**  **White** | **Hispanic:**  **White** | **Black:**  **White** | **Hispanic:**  **White** |
| 2012 | 0.51 | 0.11 | 0.13 | 0.46 | 0.13 | 0.17 | 0.22 | 0.25 | 0.28 | 0.37 |
| 2013 | 0.62 | 0.1 | 0.15 | 0.55 | 0.12 | 0.2 | 0.16 | 0.24 | 0.22 | 0.36 |
| 2014 | 1.53 | 0.17 | 0.29 | 1.37 | 0.22 | 0.39 | 0.11 | 0.19 | 0.16 | 0.28 |
| 2015 | 3.89 | 0.4 | 0.73 | 3.45 | 0.53 | 0.97 | 0.10 | 0.19 | 0.15 | 0.28 |
| 2016 | 7.29 | 0.78 | 1.38 | 6.41 | 1.03 | 1.81 | 0.11 | 0.19 | 0.16 | 0.28 |
| 2017 | 10.73 | 1.2 | 2.18 | 9.38 | 1.59 | 2.85 | 0.11 | 0.20 | 0.17 | 0.30 |
| 2018 | 15.29 | 1.8 | 3.29 | 13.26 | 2.4 | 4.28 | 0.12 | 0.22 | 0.18 | 0.32 |
| 2019 | 19.61 | 2.31 | 4.18 | 17.01 | 3.07 | 5.4 | 0.12 | 0.21 | 0.18 | 0.32 |
| 2020 | 24.85 | 3.26 | 5.92 | 21.51 | 4.3 | 7.55 | 0.13 | 0.24 | 0.20 | 0.35 |
| 2021 | 25.87 | 3.49 | 5.99 | 22.23 | 4.64 | 7.65 | 0.13 | 0.23 | 0.21 | 0.34 |

Base case estimates used the (naïve) assumption that the distribution of race/ethnicity for PrEP users without documented race/ethnicity data was the same as the distribution for those with documented race/ethnicity data.  Sensitivity analyses used published data about the characteristics of patients with initially missing race/ethnicity data to adjust our estimates.
